# Changes in Cerebrospinal Fluid Biomarkers in Human Herpesvirus-6-Associated Acute Encephalopathy/Febrile Seizures

**DOI:** 10.1155/2014/564091

**Published:** 2014-09-11

**Authors:** Naoyuki Tanuma, Rie Miyata, Keisuke Nakajima, Akihisa Okumura, Masaya Kubota, Shin-ichiro Hamano, Masaharu Hayashi

**Affiliations:** ^1^Department of Pediatrics, Tokyo Metropolitan Fuchu Medical Center for the Disabled, 2-9-2 Musashidai, Fuchu, Tokyo 183-8553, Japan; ^2^Department of Brain Development and Neural Regeneration, Tokyo Metropolitan Institute of Medical Science, Tokyo 156-8506, Japan; ^3^Department of Pediatrics, School of Medicine, Juntendo University, Tokyo 113-8421, Japan; ^4^Department of Neurology, National Center for Child Health and Development, Tokyo 157-8535, Japan; ^5^Division of Neurology, Saitama Children's Medical Center, Saitama 339-8551, Japan

## Abstract

To determine the involvement of oxidative stress in the pathogenesis of acute encephalopathy associated with human herpesvirus-6 (HHV-6) infection, we measured the levels of oxidative stress markers 8-hydroxy-2′-deoxyguanosine (8-OHdG) and hexanoyl-lysine adduct (HEL), tau protein, and cytokines in cerebrospinal fluid (CSF) obtained from patients with HHV-6-associated acute encephalopathy (HHV-6 encephalopathy) (*n* = 16) and complex febrile seizures associated with HHV-6 (HHV-6 complex FS) (*n* = 10). We also examined changes in CSF-8OHdG and CSF-HEL levels in patients with HHV-6 encephalopathy before and after treatment with edaravone, a free radical scavenger. CSF-8-OHdG levels in HHV-6 encephalopathy and HHV-6 complex FS were significantly higher than in control subjects. In contrast, CSF-HEL levels showed no significant difference between groups. The levels of total tau protein in HHV-6 encephalopathy were significantly higher than in control subjects. In six patients with HHV-6 infection (5 encephalopathy and 1 febrile seizure), the CSF-8-OHdG levels of five patients decreased after edaravone treatment. Our results suggest that oxidative DNA damage is involved in acute encephalopathy associated with HHV-6 infection.

## 1. Introduction

Viral infection-associated acute encephalopathy/encephalitis is a serious complication with neurological sequelae. The main symptoms of the acute phase are impaired consciousness and convulsive status epilepticus with hyperpyrexia. Several subtypes of acute encephalopathy have been established based on clinical, radiologic, and laboratory findings. Acute encephalopathy with biphasic seizures and late reduced diffusion (AESD) is a new subtype characterized by a prolonged febrile seizure (FS) on day 1, which usually lasts longer than 30 min, as the initial neurological symptom [1, 2]. The initial seizures are followed by secondary seizures, most often a cluster of complex partial seizures on days 4–6. Magnetic resonance imaging (MRI) shows no acute abnormalities until day 1 or 2 but reveals reduced subcortical diffusion from day 3 onwards. Hoshino et al. reported that AESD was the most frequent syndrome in a nationwide survey on the epidemiology of acute encephalopathy in Japan and that human herpesvirus-6 (HHV-6) was the most common preceding pathogenic infection in AESD [3]. Recent studies demonstrated three potential major pathomechanisms of viral associated encephalopathy: metabolic error, cytokine storm, and excitotoxicity [4]. However, the exact pathogenesis remains unknown.

Oxidative stress originates from an imbalance between the production of reactive oxygen species (ROS) and, to a lesser extent, reactive nitrogen species (RNS), and the antioxidant capacities of cells and organs [5]. Recently, oxidative stress was confirmed to play a role in adult-onset neurodegenerative diseases such as Alzheimer's disease, Parkinson's disease, and amyotrophic lateral sclerosis [6, 7]. We confirmed the involvement of oxidative neuronal damage in child-onset neurodegenerative diseases, such as subacute sclerosing panencephalitis [8], xeroderma pigmentosum [9], Cockayne syndrome [10], and spinal muscular atrophy [11].

In the present study, we measured the levels of oxidative stress markers (8-hydroxy-2′-deoxyguanosine: 8-OHdG and hexanoyl-lysine adduct: HEL), tau protein, and cytokines in cerebrospinal fluid (CSF) obtained from patients with HHV-6-associated encephalopathy and complex FS associated with HHV-6 infection.

## 2. Patients and Methods

### 2.1. Patients

We analyzed CSF obtained in the acute phase of inpatients with HHV-6-associated encephalopathy (HHV-6 encephalopathy) (*n* = 16) and complex FS associated with HHV-6 (HHV-6 complex FS) (*n* = 10) during the period from 2008 to 2010. Laboratory diagnoses of HHV-6 infection were based on a virus-specific polymerase chain reaction (PCR) assay or detection of virus-specific antibodies. Diagnosis of acute encephalopathy or complex FS was performed by the attending physician and later confirmed by examination of available clinicoradiological information. All cases of HHV-6-associated encephalopathy were diagnosed based on the clinical course and MRI findings. The complex FS group consisted of children who presented with fever and seizure but were later found to be free from acute neurological damage based on the clinical course, laboratory data, and brain imaging. Another 16 children (15 with fever but not central nervous system infection and 1 with hypoglycemia) were also enrolled as control subjects. Parent consent was obtained in all subjects in accordance with the Helsinki Declaration and all protocols were approved by the institutional ethics committee of the Tokyo Metropolitan Fuchu Medical Center for the Disabled.

### 2.2. Sample Collection and Measurement of CSF Biomarkers

CSF samples were obtained from each patient at any point during the disease and immediately stored at −80°C until they were analyzed. The amount of DNA oxidative stress marker, 8-hydroxy-2′-deoxyguanosine (8-OHdG), and the early stage lipid peroxidation marker, hexanoyl-lysine adduct (HEL), was examined using commercially available enzyme-linked immunosorbent assay (ELISA) kits (Japan Institute for the Aging, Shizuoka, Japan). Total tau protein was determined using sandwich ELISA (Invitrogen Corporation, Camarillo, CA). The levels of cytokines were evaluated by multiplex bead-based immunoassay (BioPlex 200 system) (Bio-Rad Laboratories, Inc., Hercules, CA). All assays were carried out according to the manufacturer's protocols. The detection limit for each ELISA kit was 0.06 ng/mL (8-OHdG), 2.6 ng/mL (HEL), and 15 pg/mL (total tau protein).

### 2.3. Edaravone Treatment

Edaravone (3-methyl-1-phenyl-2-pyrazolin-5-one) is a free radical scavenging drug that is clinically used in Japan for treatment of acute ischemic stroke [12, 13]. Several studies have shown that edaravone has preventive effects on brain injury following ischemia and reperfusion in patients with brain attack [14, 15]. Based on these observations, six patients with HHV-6 infection (5 patients with encephalopathy and 1 patient with complex FS) received free radical scavenger edaravone treatment in addition to conventional therapy for acute encephalopathy. A standard treatment protocol is edaravone 0.5 mg/kg every 12 hours (1 mg/kg daily) intravenously for 7–12 days. Parent consent was obtained in all patients before the treatment.

### 2.4. Statistical Analysis

Data were analyzed by GraphPad Prism version 5.0. Differences in oxidative stress markers, tau protein, and cytokine levels among each group were analyzed by one-way analysis of variance (ANOVA) and Dunn's multiple comparison test. Correlations between CSF-8OHdG and other biomarkers were evaluated using Spearman's rank correlation coefficient. We used Fisher's exact test to examine the relationship between increased levels of each biomarker and the presence or absence of neurological sequelae in HHV-6 encephalopathy. Comparisons of levels of CSF biomarkers before and after edaravone treatment were performed by paired *t*-test. A *P* value of less than 0.05 was considered statistically significant.

## 3. Results

### 3.1. Study Population and Clinical Features

The characteristics of the patients included in the study are summarized in Table 1. There were no significant differences of age among each group. Thirteen of 16 patients (81.3%) with HHV-6 encephalopathy were AESD, and only five patients (31.3%) recovered without sequelae from HHV-6 encephalopathy. In contrast, all patients with complex FS associated with HHV-6 infection were without neurological sequelae.

### 3.2. Oxidative DNA Damage and Lipid Peroxidation in HHV-6 Encephalopathy and Complex FS

**Figure 1:**
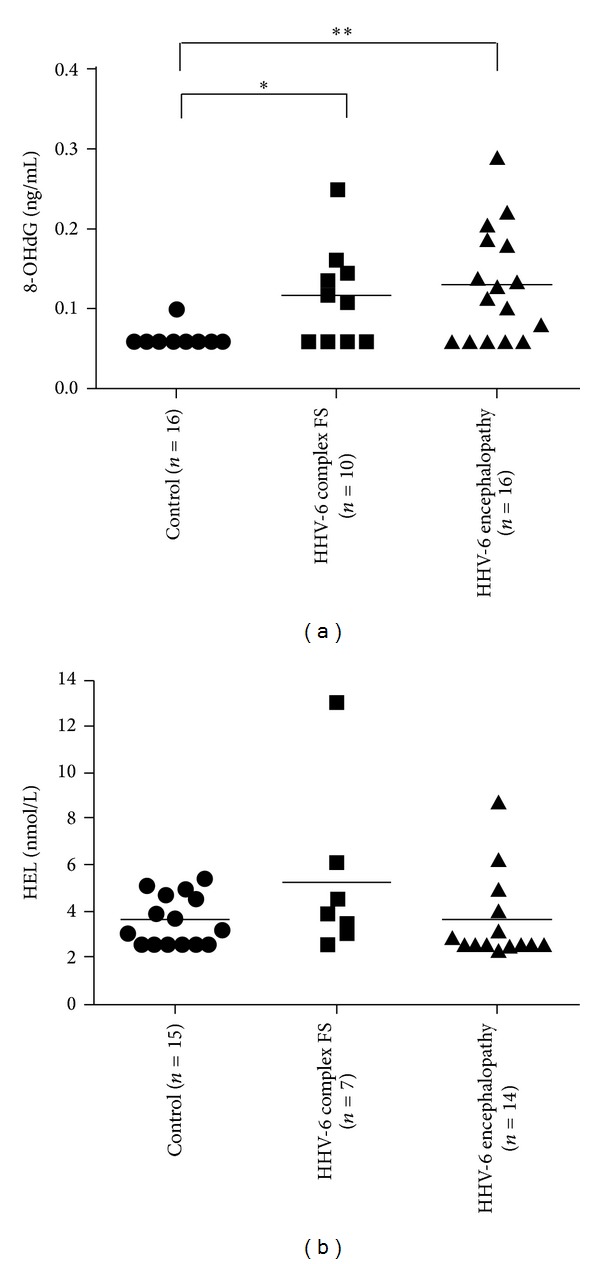
Cerebrospinal fluid (CSF) levels of oxidative stress markers in HHV-6 encephalopathy, HHV-6 complex febrile seizures (FS), and controls. (a) 8-hydroxy-2′-deoxyguanosine: 8-OHdG, (b) hexanoyl-lysine adduct: HEL. *P < 0.05, **P < 0.01. The horizontal bar indicates the mean value of each group. CSF-8OHdG levels (mean ± SD) in HHV-6 encephalopathy, HHV-6 complex FS, and controls are 0.129 ± 0.07 ng/mL, 0.116 ± 0.061 ng/mL, and 0.063 ± 0.01 ng/mL, respectively. CSF-HEL levels (mean ± SD) in HHV-6 encephalopathy, HHV-6 complex FS, and control subjects are 3.59 ± 1.87 nmol/L, 5.24 ± 3.63 nmol/L, and 3.62 ± 1.08 nmol/L, respectively.

The CSF-8-OHdG levels in HHV-6 encephalopathy (0.129 ± 0.07 ng/mL, mean ± SD, *P* < 0.01) and HHV-6 complex FS (0.116 ± 0.061 ng/mL, mean ± SD, *P* < 0.05) patients were significantly higher than in control subjects (0.063 ± 0.01 ng/mL, mean ± SD) (Figure 1(a)). CSF-HEL levels (mean ± SD) in HHV-6 encephalopathy, HHV-6 complex FS, and control subjects were 3.59 ± 1.87 nmol/L, 5.24 ± 3.63 nmol/L, and 3.62 ± 1.08 nmol/L, respectively. There were no significant differences in CSF-HEL levels between all groups (Figure 1(b)). These data are summarized in Table 2.

### 3.3. Total Tau Protein Levels in HHV-6 Encephalopathy and Complex FS

**Figure 2:**
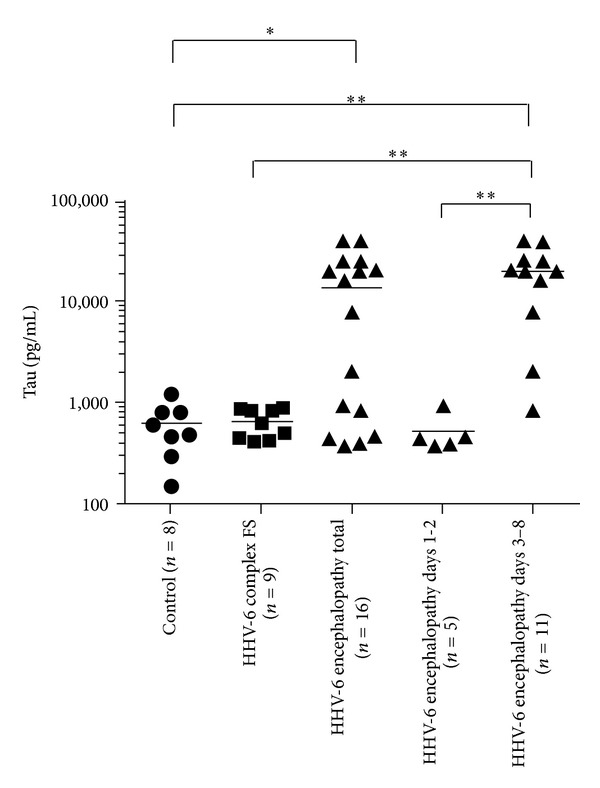
Cerebrospinal fluid (CSF) tau protein levels. The horizontal bar indicates the mean value of each group. CSF levels (mean ± SD) of tau protein in HHV-6 encephalopathy, HHV-6 complex febrile seizures (FS), and controls are 13, 905.6 ± 14, 201.1 pg/mL, 654.7 ± 213.7 pg/mL, and 609.0 ± 342.0 pg/mL, respectively. Total tau protein levels in HHV-6 encephalopathy patients are significantly higher than in control subjects (*P < 0.05). The levels of tau protein in HHV-6 encephalopathy at days 3–8 (19, 856.9 ± 13, 121.9 pg/mL) are significantly higher than those of HHV-6 encephalopathy at days 1-2 (520.4 ± 229.6 pg/mL), HHV-6 complex FS, and controls (**P < 0.01).

Total tau protein levels in HHV-6 encephalopathy patients (*n* = 16) (13, 905.6 ± 14, 201.1 pg/mL, mean ± SD) were significantly higher than in control subjects (609.0 ± 342.0 pg/mL, mean ± SD) (*P* < 0.05, Figure 2). However, there were no significant differences in CSF tau protein levels between the HHV-6 encephalopathy group and HHV-6 FS group (654.7 ± 213.7 pg/mL, mean ± SD). We then divided the HHV-6 encephalopathy group into two groups according to sampling time at days 1-2 (*n* = 5) and days 3–8 (*n* = 11), respectively. Consequently, we found that the levels of tau protein were significantly increased at days 3–8 in HHV-6 encephalopathy (19, 856.9 ± 13, 121.9 pg/mL, mean ± SD) compared with those of HHV-6 encephalopathy at days 1-2 (520.4 ± 229.6 pg/mL, mean ± SD) (*P* < 0.01), HHV-6 complex FS (*P* < 0.01), and controls (*P* < 0.01) (Figure 2).

### 3.4. CSF Cytokine Profile in Acute Encephalopathy and Complex FS

**Figure 3:**
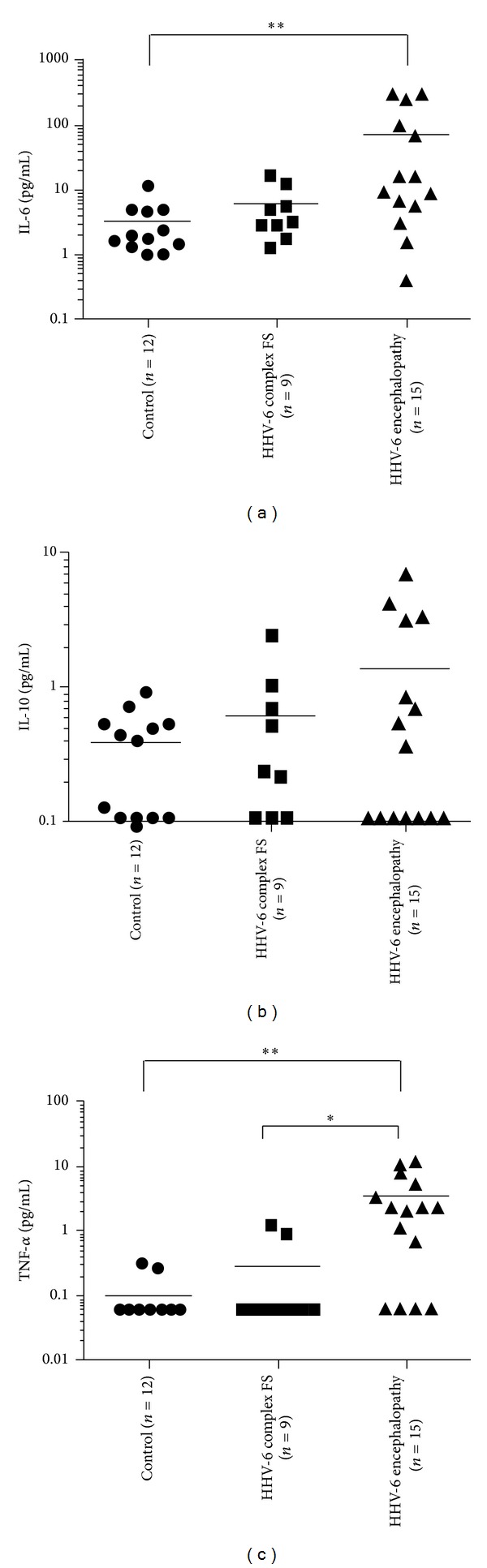
Cerebrospinal fluid (CSF) cytokine levels. The horizontal bar indicates the mean value of each group. (a) Levels of CSF IL-6 in patients with HHV-6 encephalopathy, HHV-6 febrile seizures (FS), and controls are 74.6 ± 116.9 pg/mL, 5.8 ± 5.3 pg/mL, and 3.2 ± 3.0 pg/mL, respectively. The CSF IL-6 levels in patients with HHV-6 encephalopathy are significantly higher than in controls (**P < 0.01). (b) Levels of CSF IL-10 in patients with HHV-6 encephalopathy, HHV-6 febrile seizures (FS), and controls are 1.4 ± 2.1 pg/mL, 0.6 ± 0.8 pg/mL, and 0.4 ± 0.3 pg/mL, respectively. (c) Levels of CSF TNF-*α* in patients with HHV-6 encephalopathy, HHV-6 febrile seizures (FS), and controls are 3.4 ± 4.0 pg/mL, 0.3 ± 0.5 pg/mL, and 0.1 ± 0.1 pg/mL, respectively. The CSF TNF-*α* levels in patients with HHV-6 encephalopathy are significantly higher than those with complex FS and in controls (*P < 0.05 and **P < 0.01, resp.).

We next confirmed the elevation of CSF IL-6 and TNF-*α* in patients with HHV-6 encephalopathy (Figure 3). The CSF IL-6 levels in patients with HHV-6 encephalopathy (74.6 ± 116.9 pg/mL, mean ± SD) were significantly higher than in controls (3.2 ± 3.0 pg/mL, mean ± SD) (*P* < 0.01) (Figure 3(a)). The CSF TNF-*α* levels in patients with HHV-6 encephalopathy (3.4 ± 4.0 pg/mL, mean ± SD) were also significantly higher than those with complex FS (0.3 ± 0.5 pg/mL, mean ± SD) and in controls (0.10 ± 0.1 pg/mL, mean ± SD) (*P* < 0.05 and *P* < 0.01, resp.) (Figure 3(c)). In contrast, there were no significant differences of CSF IL-10 levels among patients with HHV-6 encephalopathy or HHV-6 complex FS and controls (Figure 3(b)).

### 3.5. Correlation Analysis of CSF Biomarkers in HHV-6 Encephalopathy

We next examined correlations between CSF-8OHdG and other biomarkers in the HHV-6 encephalopathy group (Table 3). There was a significant positive correlation between IL-6 and TNF-*α* (Spearman *r* = 0.783, *P* = 0.0006). However, there were no significant correlations among other biomarkers. In addition, there was no correlation between the increased levels of each biomarker and the presence or absence of neurological sequelae in HHV-6 encephalopathy (data not shown).

### 3.6. Changes in CSF-8-OHdG and CSF-HEL Levels before and after Edaravone Treatment in HHV-6-Associated Acute Encephalopathy and Complex FS

**Figure 4:**
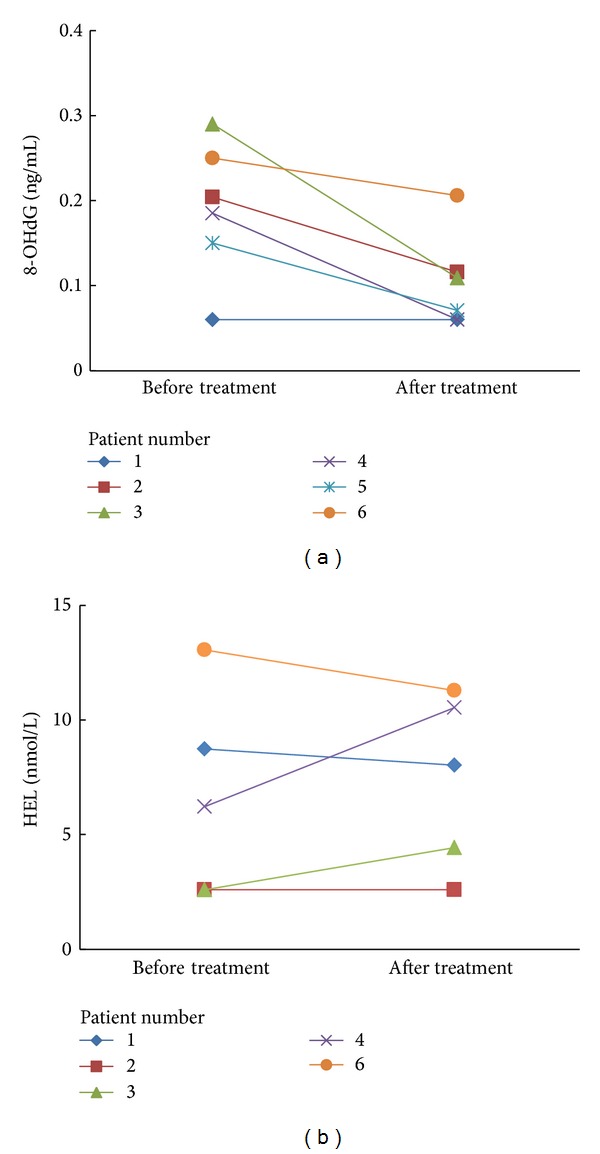
Changes in cerebrospinal fluid (CSF)-8-OHdG (a) and CSF-HEL (b) levels before and after edaravone treatment in HHV-6-associated acute encephalopathy and complex febrile seizure (FS) patients. CSF-8-OHdG levels are decreased after edaravone treatment (*P* = 0.0202, paired *t*-test).

Finally, we compared the CSF levels of oxidative stress markers in six patients with HHV-6 infection (5 patients with encephalopathy and 1 patient with febrile seizures) before and after edaravone treatment. Clinical profile of patients with edaravone treatment is shown in Table 4. The mean initiation time of edaravone treatment was day 4.8 for the HHV-6 encephalopathy group. One patient with febrile seizures associated with HHV-6 infection who received edaravone treatment from day 1 did not develop encephalopathy and recovered without sequelae (patient 6). The CSF-8-OHdG levels decreased after edaravone treatment (*P* = 0.0202, paired *t*-test) (Figure 4(a)). Regarding the CSF-HEL levels, there were no significant differences between before and after edaravone treatment. We also compared the mean CSF levels of other biomarkers before and after treatment and observed no significant differences of mean values (data not shown).

## 4. Discussion

In the present study, we demonstrated that CSF-8-OHdG levels in HHV-6 encephalopathy and HHV-6 complex FS patients were significantly higher than in controls, suggesting increased oxidative stress is induced by HHV-6 infection. Recent studies revealed that oxidative damage is an emerging general mechanism of nervous system injury caused by viral infection. For example, oxidative injury is a component of acute encephalitis caused by herpes simplex virus type 1 (HSV-1) [16]. HSV-1 infection of nervous system tissues in mice was associated with the expression of inducible nitric oxide synthase (iNOS) and the release of cytokines including TNF-*α* from inflammatory cells. Thus, increased generation of ROS and RNS can be caused by the direct effects of virus on cells and the indirect effects of host inflammatory responses [17]. Regarding HHV-6 infection, Fukuda et al. reported that urinary 8-OHdG concentrations in a patient with HHV-6 encephalopathy on the first day of hospitalization were 1.5 times higher than the mean concentration in healthy children and they peaked at the second seizures [18]. They speculated that 8-OHdG was produced by ROS from cytokines associated with inflammation and apoptosis following brain edema because changes in urinary 8-OHdG levels reflected the degree of brain edema. However, we found that increased levels of 8-OHdG were observed not only in HHV-6 encephalopathy but also in complex FS associated with HHV-6 infection. We also showed that CSF IL-6 and TNF-*α* levels were elevated only in the HHV-6 encephalopathy group, but not in the HHV-6 complex FS group. In addition, we analyzed correlations among biomarkers and observed no significant correlations between increased 8-OHdG levels and cytokine production or increased tau levels. These results suggest that oxidative DNA damage in the brain caused by HHV-6 infection may be independent of inflammatory reactions and subsequent axonal damage.

In contrast with the increased levels of 8-OHdG, there was no significant increase of CSF-HEL levels in HHV-6 encephalopathy compared with HHV-6 complex FS and controls. We previously demonstrated that oxidative stress of DNA contributes to early neuronal damage, whereas lipid peroxidation is related to subsequent neurodegeneration in subacute sclerosing panencephalitis [8]. In the present study, we only examined levels of CSF biomarkers in the acute phase of the diseases. Further investigation is required to clarify whether lipid peroxidation may be involved in the chronic phase.

Most patients with HHV-6 encephalopathy in this study (81.3%) were AESD, which has a high incidence of neurological sequelae. We previously indicated that levels of CSF tau protein were elevated in patients with AESD [19]. The current study demonstrates that CSF levels of tau protein were significantly increased at days 3–8 in HHV-6 encephalopathy compared with those of HHV-6 encephalopathy at days 1-2, HHV-6 complex FS, and controls. As CSF tau protein is considered a useful biomarker of axonal damage [20], our results raise the possibility that the high incidence of neurological sequelae in AESD is attributable to axonal injury. However, there was no correlation between the increased levels of tau protein and the presence or absence of neurological sequelae. These findings suggest that tau protein is a sensitive biomarker that might help diagnose HHV-6 encephalopathy, but it is difficult to make an early diagnosis for acute encephalopathy using this biomarker. In terms of the prognostic prediction for HHV-6 encephalopathy, another biomarker will be required because increased levels of tau protein do not always reflect a poor prognosis.

Edaravone is a free radical scavenger that interacts biochemically with a wide range of free radicals [21]. In experimental models, edaravone protects against apoptotic neuronal cell death and improves cerebral function after traumatic brain injury (TBI) [21]. In addition, Ohta et al. reported that administration of edaravone to mice immediately after TBI suppressed traumatic axonal injury and oxidative stress, which protected against trauma-induced memory deficits [22]. Edaravone is used clinically in Japan for the treatment of acute ischemic stroke. Although childhood ischemic stroke is different than in adults, the use of edaravone was recently approved for the treatment of stroke in children. In the present study, we reported 5 cases of edaravone treatment for HHV-6-associated acute encephalopathy and one case of HHV-6 complex FS. Our study is very preliminary and it is likely that the efficacy of edaravone treatment in combination with other therapies at this time was poor.

There were several limitations in this study. First, the initiation of edaravone treatment was delayed in HHV-6 encephalopathy because it is difficult to distinguish HHV-6 encephalopathy from HHV-6 complex FS during the initial seizures. Early diagnosis of HHV-6 encephalopathy, especially AESD, will be required to overcome this problem. Second, a clinical trial of edaravone for the treatment of acute encephalopathy might be difficult ethically, as placebo control cannot be used because of the severe nature of this disease. We confirmed that CSF-8-OHdG levels decreased after edaravone treatment, although there were no significant differences of mean values of other biomarkers between before and after the treatment. These results suggest edaravone treatment was partially effective for HHV-6 encephalopathy. Although these findings are encouraging, the therapeutic implications of ROS and RNS scavengers are complex, owing to their potential to exert toxic as well as protective effects [23].

## 5. Conclusion

In summary, we found oxidative DNA damage is involved in acute encephalopathy/febrile seizures associated with HHV-6 infection and may be independent of inflammatory reactions and subsequent axonal damage.

## Figures and Tables

**Table 1:** Clinical characteristics of HHV-6 encephalopathy and febrile seizure patients.

	HHV-6 encephalopathy	HHV-6 complex febrile seizures	Controls
Number of patients	16	10	16
Age (months)	15.1 ± 5.4	12.6 ± 3.9	11.1 ± 10.8
Sex ratio (M : F)	8 : 8	5 : 5	11 : 5
Sampling time (day of illness)	1–8	1	—
MRI abnormality	14/16	ND	ND
Outcome (without sequelae)	5/16	10/10	—

HHV: human herpesvirus; No.: number; ND: not done; M: male; F: female; MRI: magnetic resonance imaging.

**Table 2:** Descriptive statistics for the biomarkers examined^a^.

Biomarkers	Controls	HHV-6 complex FS	HHV-6 encephalopathy	*P*			
				Global	Controls versus HHV-6 FS	Controls versus HHV-6 encephalopathy	HHV-6 FS versus HHV-6 encephalopathy
8-OHdG, ng/mL	0.063 (0.01)	0.116 (0.061)	0.129 (0.07)	0.0025	<0.05	<0.01	ns
HEL, nmol/L	3.62 (1.08)	5.24 (3.63)	3.59 (1.87)	0.1863	ns	ns	ns
Tau, pg/mL	609.0 (342.0)	654.7 (213.7)	13,905.6 (14,201.1)	0.0028	ns	<0.05	ns
IL-6, pg/mL	3.2 (3.0)	5.8 (5.3)	74.6 (116.9)	0.0349	ns	<0.01	ns
IL-10, pg/mL	0.4 (0.3)	0.6 (0.8)	1.4 (2.1)	0.1663	ns	ns	ns
TNF-*α*, pg/mL	0.1 (0.1)	0.3 (0.5)	3.4 (4.0)	0.0036	ns	<0.01	<0.05

8-OHdG: 8-hydroxy-2′-deoxyguanosine; HEL: hexanoyl-lysine adduct; HHV-6: human herpesvirus-6; FS: febrile seizure; ns: not significant; IL: interleukin; TNF: tumor necrosis factor.

^a^Values are expressed as the mean (standard deviation).

**Table 3:** Correlation analysis of CSF biomarkers in HHV-6 encephalopathy.

Biomarker		8-OHdG	HEL	Tau	IL-6	IL-10	TNF-*α*
8-OHdG	Spearman *r*	1.000	−0.292	−0.239	−0.484	−0.046	−0.315
	*P*	<0.0001	0.312	0.373	0.068	0.871	0.253

HEL	Spearman *r*	−0.292	1.000	0.277	0.476	−0.286	0.497
	*P*	0.312	<0.0001	0.338	0.086	0.322	0.070

Tau	Spearman *r*	−0.239	0.277	1.000	−0.036	−0.224	0.091
	*P*	0.373	0.338	<0.0001	0.899	0.422	0.748

IL-6	Spearman *r*	−0.484	0.476	−0.036	1.000	0.226	0.783
	*P*	0.068	0.086	0.899	<0.0001	0.418	0.0006

Il-10	Spearman *r*	−0.046	−0.286	−0.224	0.226	1.000	0.166
	*P*	0.871	0.322	0.422	0.418	<0.0001	0.555

TNF-*α*	Spearman *r*	−0.315	0.497	0.091	0.783	0.166	1.000
	*P*	0.253	0.070	0.748	0.0006	0.555	<0.0001

CSF: cerebrospinal fluid; HHV: human herpesvirus; 8-OHdG: 8-hydroxy-2′-deoxyguanosine; HEL: hexanoyl-lysine adduct; IL: interleukin; TNF: tumor necrosis factor.

**Table 4:** Clinical profile of patients receiving edaravone treatment.

Patient	Clinical diagnosis	Age/sex	Initiation and dosage of edaravone treatment	Other treatments	Outcomes
1	HHV-6 encephalopathy	14 m/M	Day 5 0.5 mg/kg × 2/day × 7 days	Mannitol, Dexamethasone, Ganciclovir, MDL, PHT	Intellectual disability

2	HHV-6 encephalopathy (AESD)	12 m/F	Day 5 0.5 mg/kg × 2/day × 8 days	Mannitol, Dexamethasone, Ganciclovir, DZP, MDL	Without sequelae

3	HHV-6 encephalopathy	14 m/M	Day 4 0.5 mg/kg × 2/day × 10 days	Mannitol, Dexamethasone, Ganciclovir/acyclovir, DZP, MDL	Hemophagocytic syndrome Died of fulminant hepatitis

4	HHV-6 encephalopathy (AESD)	20 m/F	Day 7 0.5 mg/kg × 2/day × 7 days	Mannitol, Dexamethasone, Aciclovir, MDL	Lt hemiparesis

5	HHV-6 encephalopathy (AESD)	12 m/M	Day 3 15 mg/day × 10 days	DZP, MDL, steroid pulse therapy, mild therapeutic hypothermia	Moderate psychomotor retardation

6	HHV-6 febrile seizures	10 m/F	Day 1 0.5 mg/kg × 2/day × 12 days	Mannitol, Dexamethasone, Ganciclovir/acyclovir, DZP, MDL, PHT	Without sequelae

m: months; M: male; F: female; DZP: diazepam; MDL: midazolam; PHT: phenytoin; Lt: left; HHV: human herpesvirus; AESD: acute encephalopathy with biphasic seizures and late reduced diffusion.
